# Prevalence of Chemosensitive Neurological Disorders of Smell and Taste and Association with Blood Groups in SARS-CoV-2 Patients: Cross-Sectional Study

**DOI:** 10.3390/v15061277

**Published:** 2023-05-30

**Authors:** Samia T. Al-Shouli, Sultan Ayoub Meo, Nouf O. Alafaleq, Khalid M. Sumaily, Aseel Alshehri, Abdulrahman Almutairi, Azaam Eidalsharif, Fahad Alsulami, Saad Alhanaya

**Affiliations:** 1 Immunology Unit, Department of Pathology, College of Medicine, King Saud University, Riyadh 11461, Saudi Arabia; 2 Department of Physiology, College of Medicine, King Saud University, Riyadh 11461, Saudi Arabia; 3 Department of Biochemistry, College of Science, King Saud University, Riyadh 11461, Saudi Arabia; 4 Clinical Biochemistry Unit, Department of Pathology, College of Medicine, King Saud University, Riyadh 11461, Saudi Arabia; 5 College of Medicine, King Saud University, Riyadh 11461, Saudi Arabia

**Keywords:** SARS-CoV-2, COVID-19, prevalence, anosmia, ageusia, blood groups

## Abstract

The Severe Acute Respiratory Syndrome Coronavirus 2 (SARS-CoV-2) has caused a highly challenging and threatening situation worldwide. SARS-CoV-2 patients develop various clinical symptoms. The olfactory and taste dysfunctions are potential neurological manifestations among SARS-CoV-2 patients; however, their relationship with blood groups has rarely been investigated. This study aimed to investigate the prevalence of chemosensitive neurological disorders of smell and taste and their association with blood groups in SARS-CoV-2 patients. The present cross-sectional study was performed in the Department of Pathology, and Physiology, College of Medicine, King Saud University, Riyadh, Saudi Arabia. A well-structured, self-administered questionnaire was designed and distributed through social media platforms. A total of 922 Saudi and non-Saudi adults aged 18 years or older participated in the study. Out of 922 participants, the number of people who had anosmia was 309 (33.5%), 211 (22.9%) had hyposmia, and 45 (4.8%) had dysosmia. Moreover, 180 (19.52%) had ageusia, 47 (5.1%) and 293 (31.8%) had hypogeusia and dysgeusia, respectively. Among all the participants, 565 (61.27%) had smell-related disorders and 520 (56.39%) participants had taste-related clinical symptoms. The occurrence of anosmia and ageusia was relatively high among females compared to males (*p* = 0.024). The prevalence of smell-related disorders was 25.0% (230) and taste-related disorders was 23.21% (214) among the study participants with blood group O compared to all other blood group (A, B, and AB) participants who have smell allied disorders 30.69% (283), and taste allied disorders 27.98% (258). The prevalence of chemosensitive neurological disorders involving impaired smell and taste was higher in SARS-CoV-2 patients. These clinical symptoms were common among the participants with blood group type O compared to all other ABO blood group types. The role of certain demographic characteristics was consistent throughout multiple studies, notably with female gender and young adults.

## 1. Introduction

The outbreak of Severe Acute Respiratory Syndrome Coronavirus 2 (SARS-CoV-2) was initiated in Wuhan City, Hubei, China, in late 2019. The outbreak caused a worldwide pandemic known as COVID-19, which forced the world into a period of lockdown with a considerable impact on public health, socio-psychological well-being, and immense economic damage due to mass quarantines and mortality [1,2]. SARS-CoV-2 cases are still reported worldwide; however, due to massive efforts of the global community and vaccine campaigns, the prevalence of cases and deaths has been minimized [2]. As of 21 March 2023, worldwide, there were 761,071,826 cases and 6,879,677 deaths, and 13,259,928,837 vaccine doses administered [3]. The World Health Organization (WHO) emphasized maintaining the momentum for increasing access to COVID-19 vaccines and accelerating vaccine delivery, to save lives. The health officials continue to work towards vaccinating their populations, prioritizing the vaccination of 100% of health workers and most vulnerable groups [3].

Even with huge global efforts to end the pandemic, fully vaccinated individuals can be infected and experience various complications including neurological complications regardless of their vaccination status [3]. SARS-CoV-2 can induce anosmia and ageusia at high levels in younger patient [4]. A study conducted in Spain in March 2022 reported that blood group O may specifically increase susceptibility to COVID-19 with increased odds of having post-COVID-19 symptoms. Furthermore, the number of symptoms in each subject and the biochemical tests showed that blood group O subjects had a 4-fold higher risk of post-COVID-19 clinical symptoms compared to the non-O blood groups [5,6].

In studies concerning COVID-19, the prevalence of anosmia and ageusia in Saudi Arabia were conducted previously; however, few tried to incorporate ABO blood groups into their study, and even globally there is a profound scarcity regarding this topic. One such example was conducted in Pakistan which found that patients with the blood group B were the most likely group to become infected [7]. Variations of the severity of anosmia were reported; a study conducted in October 2020 found that 24% of patients in the study reported having mild hyposmia, 13% had moderate hyposmia, 30% had severe hyposmia, and 32% had anosmia [8]. The literature highlights the relationship of SARS-CoV-2 infection with various clinical features. The olfactory and taste dysfunctions are potential neurological manifestations among SARS-CoV-2 patients; however, their relationship with blood groups has rarely been investigated. This study aimed to investigate the prevalence of chemosensitive neurological disorders of smell and taste and their association with blood groups in SARS-CoV-2 patients.

## 2. Subjects and Methods

### 2.1. Study Design and Settings

This cross-sectional study was conducted in the Department of Pathology and Physiology, College of Medicine, King Saud University, Riyadh, Saudi Arabia, during the period of August 2022 to January 2023.

### 2.2. Questionnaire Development

The research team members designed the questionnaire and distributed it as a pilot study among the ten faculty members to check the technical concerns in the questionnaire. The questionnaire was prepared in simple language, and was easy to understand and short in length. It was also explained that the data would only be used for research purposes. Moreover, the identity of the contributors was kept confidential, and participants were able to leave the study at any stage. A bilingual (Arabic and English) self-administered computer-based questionnaire was designed and distributed via a link survey to different social media platforms, including emails, WhatsApp, and Instagram, and users were encouraged to share the questionnaire with others. Therefore, the convenience sampling technique was used.

### 2.3. Sample Size

The study participants were residents of Saudi Arabia, male and female Saudi and Non-Saudi, aged 18 or older. The sample size was calculated, based on a 50% population proportion, with a 95% confidence interval and 5% margin of error; the sample size was calculated using the formula *n* = Z^2^ P(1 − P)/d^2^, and the results were multiplied by 2 and a 20% non-response rate was added. For this study, a sample size of 922 was needed. The study inclusion criteria were participants who are 18 years or older, residents of Saudi Arabia, and have been infected with SARS-CoV-2. We excluded participants who were below the age of 18, residents of other countries, or had not been infected with SARS-CoV-2.

### 2.4. The Study Variables

The study variables were the socio-demographic characteristics of age, gender, blood type, history of COVID-19 infection, chronic diseases, vaccine doses, and clinical symptoms. The outcome variables were anosmia, hyposmia, dysosmia, ageusia, hypogeusia, and dysgeusia.

### 2.5. Data Analysis

The data were analyzed using statistical software for Windows version 26.0 (IBM, New York, NY, USA). The descriptive frequencies and percentages were used to describe the categorical and quantitative variables. The Student’s *t*-test was employed for independent samples; one-way analysis of variance followed by a post hoc test was used for quantitative outcome variables. Pearson’s Chi-square test was used to test the link between the categorical variables. The Chi-square test was not applicable; hence, an alternative Fisher’s exact test was used, and a *p*-value less than 0.05 was considered statistically significant.

### 2.6. Ethical Approval

This study was performed after approval from the Institutional Review Board, College of Medicine, King Saud University, Riyadh, Saudi Arabia (22/0613/IRB).

## 3. Results

A total of 1461 participants completed the questionnaire; the participants with a history of SARS-CoV-2 were included resulting in a total of 922 (63.1%) participants of which 561 (60.8%) were males and 361 (39.2%) were females. The age groups of the participants were as follows: 18 to 29 years (501; 54.3%), 30 to 49 years (331; 35.9%), 50 to 59 years (66; 7.2%), and 60 and above (24; 2.6%). Most of the participants were Saudis (824; 89.4%) from the central region (598; 64.85%). The most common blood type was O + ve (366; 39.7%), with non-O blood groups (A, B, and AB) accounted for 556 (60.3%) participants. The demographic characteristics of the participants are summarized in Table 1.

Table 2 shows that among the participants, 905 (98.2%) received the COVID-19 vaccine; the majority had one dose (72.10%), 19.3% received two doses, 2.5% received three, 4.2% received four, and 1.8% did not receive any vaccine. While analyzing the chronic conditions, 732 (79.4%) participants were found to be free of any chronic diseases. The common symptoms of SARS-CoV-2 infection other than anosmia/hyposmia and ageusia/hypogeusia included fever (75.6%), cough (50.9%), fatigue (56.8%), myalgia (54.4%), and headache (52.3%). As for hospitalization, 80 (8.7%) of the participants needed it.

Table 3 demonstrates the impact of SARS-CoV-2 infection on the sense of smell and taste. A complete loss of smell was seen in 33.5% of the participants, a decrease was seen in 22.9%, and an abnormal change in 4.9% of participants. In comparison, a complete loss of taste was seen in 19.5% of the participants, a decrease was seen in 5.1%, and an abnormal change in 31.8% of participants. The sense of smell and taste were not affected in 38.7% and 43.6% of participants, respectively. Looking at the recovery period of those whose sense of smell was affected, 48.6% took less than a month to recover, 8.8% took 2–6 months, and 2.8% took more than 6 months to recover. Amongst those whose sense of taste was affected, 51.8% took less than a month to recover, 7.8% took 2–6 months, and 2.3% took more than 6 months to recover. Of those whose sense of smell and taste were affected, 5.2% and 3.6% of participants did not recover, respectively.

Regarding the statistically significant associations, it appears that patients who are blood group O were more likely to develop smell disturbances as well as taste disturbances compared to all other blood group participants including blood group A, B, and AB (Table 4 and Table 5). A significant relationship (*p* < 0.05) was noted with the variables age, gender, vaccination status, as well as blood group O. It was seen that patients in the age range of 18–29 had a noticeably higher chance of experiencing anosmia (*p* < 0.001). Interestingly, it appeared that no matter the vaccination status, hyposmia was always more common than anosmia, with patients who took four doses being the most likely to develop it. Furthermore, it was noted that women were statistically more likely to develop smell complications than men (*p* = 0.024).

In general, dysgeusia was found to be the most common disorder with regard to taste being affected, and the most susceptible age range was 30–49 (Table 4). In terms of vaccination status, patients who received three doses were the least affected (*p* = 0.028) and women were affected more than men (*p* < 0.001).

## 4. Discussion

This study was performed to investigate the occurrence of chemosensitive neurological disorders of smell and taste and their association with blood groups in SARS-CoV-2 patients in Saudi Arabia using a web-based, self-administered questionnaire. Among all the participants, 61.27% had smell-related disorders and 56.39% participants had taste-related clinical symptoms. The prevalence of anosmia and ageusia was relatively higher among females compared to males. Moreover, the prevalence of smell-related disorders was 25.0% and taste-related disorders was 23.21% among the participants with blood group O which was higher than those of all the other blood groups (A, B, and AB) participants.

The present study findings are mostly consistent with studies conducted in the United States and Saudi Arabia. A systematic meta-analysis reported the prevalence of anosmia and ageusia as 52.73% and 43.93%, respectively [5]. Moreover, the Saudi study found that anosmia and ageusia were more likely to develop in females (67% and 69.5%, respectively). In addition, they found that participants in their third (44%) and fourth decades (22.2%) were more affected compared to other age groups [9]. SARS-CoV-2 can induce anosmia and ageusia at more than 10- and 8-fold higher rates compared to other respiratory or COVID-19-like diseases [9]. Our findings regarding the duration of symptoms were similar to a study conducted in Brazil; they found that the incidence of anosmia and ageusia was 18.8% with a mean time of 67 days for the former and 14.1% over a mean time of 60.7 days for the latter [7]. Meanwhile, in our study, we found that 12.7% had anosmia in a similar time frame. However, there was a difference in ageusia as we found that only 4.6% had ageusia in that time frame. Regarding vaccination status, a study conducted in Italy reported the incidence of anosmia and ageusia to be 62.3% and 53.6%, respectively, after complete vaccination [10]; this is consistent with our findings, implying that vaccination had no significant impact on the prevalence of anosmia and ageusia.

Regarding ABO blood types, there were contradictory findings amongst different studies. A study from Pakistan reported a lower occurrence of anosmia or ageusia with blood group O (30.0%) compared to blood group B (39.5%) and lower chances of showing severe symptoms of COVID-19 overall [11]. A study conducted in Spain concluded otherwise; they found that group O had an increased susceptibility to more severe symptoms [5]. In contrast, our findings were consistent with the Pakistani study, as we found that the prevalence of anosmia in blood group O was 57.1% and ageusia 53.1% which was higher than that of all other non-O blood groups including blood groups A, B, and AB. Overall, anosmia and ageusia are prevalent symptoms of COVID-19, and therefore they can be used for a somewhat accurate diagnosis. The study has some limitations. Time was restricted by a deadline. The study did not take immune and allergic conditions into account as they might play a role in the prevalence of anosmia and ageusia. In addition to being affected by selection and recall bias, the data were collected via a web-based survey.

Alabsi et al., 2022 [12] reported that the association between ABO blood groups and infectious diseases is not just a causal relationship; however, the literature demonstrates that blood groups have an impact on disease courses and prognosis. The authors evaluated the olfactory and gustatory disturbances and found that 62% of the patients had olfactory dysfunction, with about 14% having ageusia and 68% had some form of taste alteration. The blood group is a predisposing factor for smell variations in post-SARS-CoV-2 patients.

Al-Youha et al., 2021 [13] reported that ABO blood groups have been connected to susceptibility to illness with microorganisms, including coronaviruses. This study’s findings favor the hypothesis of the involvement of the ABO blood group system in predisposing to infection with SARS-CoV-2.

Shesha et al., 2022 [14] explored the link between SARS-CoV-2 infection and ABO blood groups. The frequency of blood group O + ve was 37%, A + ve was 29.20%, and B + ve was 22.6%. Jawdat et al., 2022 [15] studied ABO type and its link with SARS-CoV-2. The study outcomes offered evidence that “blood group B is a risk factor for COVID-19 disease while blood group O is protected from COVID-19 infection”. Ray et al., 2021 [16] determined the relationship between ABO and Rh blood groups and the risk for SARS-CoV-2 illness. The authors found that blood groups O and Rh- had a slightly lower risk for SARS-CoV-2 illness [16]. In another study, Göker et al., 2020 [17] reported that blood group A might have a role in higher susceptibility to COVID-19, and blood group O has a relatively protective role. Rahim et al., 2021 [18] demonstrated a link between blood groups B and AB and susceptibility to COVID-19. Similarly, Deschasaux-Tanguy et al., 2023 [19] demonstrated the dynamics of SARS-CoV-2 infection and various ABO blood groups. The authors provided an additional understanding of the SARS-CoV-2 infection, with a higher susceptibility of infection among people with blood groups A and AB and a lower risk for those with blood group O.

Since the discovery of ABO blood group types in 1901 [20], the association between ABO blood groups and various diseases has been emphasized in the literature. ABO blood groups are biologically linked to some diseases and tumorigenesis. The possible pleiotropic impact of the ABO gene over the receptor gene could be considered in the framework of these hypotheses [21,22,23]. During the severe acute respiratory syndrome coronavirus (SARS-CoV-1) epidemic, several observations suggested that ABO type may contribute to disease, with less susceptibility in individuals with group O [24]. Similarly, a relationship was also identified for SARS-CoV-2. The literature identified a high proportion of patients with group A and a lower proportion of those with Group O among COVID-19 patients [25].

In the scientific literature, numerous hypotheses describe the differences in SARS-CoV-2 infection by ABO blood types. The growing pieces of evidence from the available published scientific literature demonstrate that the ABO blood group plays a part in the pathogenesis of SARS-CoV-2 infections. Blood group A confers greater sensitivity to disease and its serious outcomes. However, people with blood group O are less likely to suffer from the SARS-CoV-2 disease. The mechanism behind the lower occurrence of SARS-CoV-2 infections among the individuals with blood group O is that anti-A and/or anti-B antibodies present in group O individuals could bind to corresponding antigens on the viral envelope and contribute to viral neutralization, thus preventing target cell infection. The SARS-CoV-2 virus and SARS-CoV spike (S) proteins may be bound by anti-A isoagglutinin in group O and group B individuals, which may block interactions between the virus and the angiotensin-converting enzyme 2 receptor to prevent viral entry into lung epithelial cells. Blood group O may be associated with a lower risk of SARS-CoV-2 infection and group A may be associated with a higher risk of SARS-CoV-2 infection along with severe clinical symptoms of the disease. Even after all these facts, in the future, studies with large sample sizes are needed to verify these relationships. Based on the available literature, data is still lacking in establishing guiding policies [26].

Olfactory and gustatory disorders are predominant characteristic clinical symptoms of SARS-CoV-2 disease [27]. It is also an established fact that the frequencies and pathogeneses are constantly changing due to swift mutations of the viral strains. Angiotensin-converting enzyme 2, a receptor for the spike protein of SARS-CoV-2 in the olfactory epithelium, is involved in the development of olfactory dysfunction. In general, olfactory dysfunctions resolve in a few weeks. However, in some patients, these clinical symptoms persist for several months, and it affects the quality of life and the patient’s oral health. It is also acknowledged that the damage due to COVID-19 encompasses olfactory nerve cells, resulting in sensorineural olfactory dysfunction [28].

In the worldwide scientific literature, similar findings have been reported showing that the blood group is a predisposing factor for smell dysfunctions in post-COVID-19 patients. It is also a fact that the continuing olfactory and gustatory chemosensory impairment affects the quality of life in general and oral health. The literature often demonstrates that blood groups have a direct impact on the course of the disease and its prognosis. The ABO blood groups are an influencing factor for persistent anosmia after COVID-19 infection. Mahmud et al. [29] demonstrated that the occurrence of SARS-CoV-2 disease in patients with blood group A was higher and there was no association of ABO blood groups with the clinical presentation or duration of recovery from COVID-19. Badedi et al. [30] did not find any significant association between a specific ABO blood group and mortality risk in COVID-19 patients. Olfactory and gustatory disorders were found in patients after COVID-19. Blood group is a predisposing factor for persistent smell alterations in post-COVID-19 patients.

The hypotheses linking the ABO blood types to SARS-CoV-2 and neurological clinical outcomes including smell- and taste-related disorders also need further studies for clarification. The possible role of ABO blood group antigens in regulating virus–cell interactions may depend on various biological factors. Further global large data-based studies are needed to understand the molecular mechanisms by which blood groups engender susceptibility to SARS-CoV-2 infections and taste and smell disorders and to develop preventive measures against viral infection and illness.

## 5. Study Strengths and Limitations

This study’s strength is that it is a novel study aimed to investigate the prevalence of chemosensitive neurological disorders of smell and taste and their association with blood groups in SARS-CoV-2 patients. Understanding the susceptibility to SARS-CoV-2 infection due to ABO blood groups and their association with taste and smell disorders could help physicians to identify the risk factors while utilizing the physiological protective factors associated with the ABO blood group. The limitation of this study is that the conclusions are based on a small sample size and hence, future studies with larger sample sizes are required to identify SARS-CoV-2 variants and susceptibility according to the ABO blood groups.

## 6. Conclusions

The prevalence of chemosensitive neurological symptoms of loss of smell and taste was more frequent in SARS-CoV-2 patients. These clinical symptoms were common among the participants with blood group type O compared to all other ABO blood group types. Certain demographic characteristics were consistent throughout multiple studies, notably with female gender and young adults. The role of blood group antigens in the pathogenesis of SARS-CoV-2 remains a fascinating subject for clinicians, policymakers, and for prognostic purposes to reduce the global burden of the disease.

## Figures and Tables

**Table 1:** Socio-demographic characteristics of the study participants (n = 922).

Socio-Demographic Characteristics	Number and %
Gender	
Male	561 (60.8)
Female	361 (39.2)
Age	
18–29	501 (54.3)
30–49	331 (35.9)
50–59	66 (7.2)
60 and above	24 (2.6)
Nationality	
Saudi	824 (89.4)
Non-Saudi	98 (10.6)
Region	
Northern Region	28 (3.03)
Eastern Region	54 (5.85)
Western Region	208 (22.55)
Middle Region	598 (64.85)
Southern Region	34 (3.68)

**Table 2:** Blood group types of study participants (n = 922).

Blood Group Type	Number and Percentage
A + ve	226 (24.5)
A − ve	25 (2.7)
B + ve	131 (14.2)
B − ve	14 (1.5)
AB + ve	49 (5.3)
AB − ve	2 (0.2)
O + ve	366 (39.7)
O − ve	37 (4)
Unknown	72 (7.8)

**Table 3:** Prevalence and duration of anosmia/hyposmia and ageusia/hypogeusia (n = 922).

Questions	Yes, Completely Lost n (%)	Yes, Decreased n (%)	Yes, Abnormally Changed n (%)	No n (%)
Was your sense of smell affected?	309 (33.5)	211 (22.9)	45 (4.9)	357 (38.7)
Was your sense of taste affected?	180 (19.5)	47 (5.1)	293 (31.8)	402 (43.6)
Duration	<1 month	2–6 months	>6 months	Not recovered
How long did it take for your sense of smell to recover?	448 (48.6)	81 (8.8)	26 (2.8)	48 (5.2)
How long did it take for your sense of taste to recover?	478 (51.8)	72 (7.8)	21 (2.3)	33 (3.6)

**Table 4:** Association between demographic variables, blood groups, and loss of sense of smell.

Variable	Was Your Sense of Smell Affected?				
	Anosmia N (%)	Hyposmia N (%)	Dysosmia N (%)	No N (%)	*p*-Value
Gender					0.024
Male	180 (32.1)	134 (23.9)	19 (3.4)	228 (40.6)	
Female	129 (35.7)	77 (21.3)	26 (7.2)	129 (35.7)	
Age					<0.001
18–29	138 (27.5)	146 (29.1)	27 (5.4)	190 (37.9)	
30–49	52 (15.7)	141 (42.6)	11 (3.3)	127 (38.4)	
50–59	15 (22.7)	17 (25.8)	3 (4.5)	31 (47.0)	
60+	6 (25.0)	5 (20.8)	4 (16.7)	9 (37.5)	
Nationality					0.137
Saudi	181 (22.0)	274 (33.3)	41 (5.0)	328 (39.8)	
Non-Saudi	30 (30.6)	35 (35.7)	4 (4.1)	29 (29.6)	
Region					0.132
Northern	2 (9.5)	12 (57.1)	0 (0.0)	7 (33.3)	
Eastern	10 (18.5)	15 (27.8)	3 (5.6)	26 (48.1)	
Western	59 (28.4)	64 (30.8)	12 (5.8)	73 (35.1)	
Middle	132 (21.8)	202 (33.4)	28 (4.6)	243 (40.2)	
Southern	8 (23.5)	16 (47.1)	2 (5.9)	8 (23.5)	
Blood type					0.05
O	124 (30.8)	87 (21.6)	19 (4.7)	173 (42.9)	
Non-O	162 (36.2)	102 (22.8)	19 (4.3)	164 (36.7)	
Unknown	23 (31.9)	22 (30.6)	7 (9.7)	20 (27.8)	
Chronic disease					0.33
Yes	45 (23.7)	67 (35.3)	13 (6.8)	65 (34.2)	
No	166 (22.7)	242 (33.1)	32 (4.4)	292 (39.9)	
Vaccine doses					0.01
One dose	155 (23.3)	207 (31.1)	33 (5.0)	270 (40.6)	
Two doses	43 (24.2)	64 (36.0)	12 (6.7)	59 (33.1)	
Three doses	9 (39.1)	9 (39.1)	0 (0.0)	5 (21.7)	
Four doses	2 (5.1)	22 (56.4)	0 (0.0)	15 (38.5)	
None	2 (11.8)	7 (41.2)	0 (0.0)	8 (47.1)	

**Table 5:** Association between demographic variables, blood groups, and loss of sense of taste.

Variable	Was Your Sense of Taste Affected?				
	Ageusia N (%)	Hypogeusia N (%)	Dysgeusia N (%)	No N (%)	*p*-Value
Gender					<0.001
Male	15 (2.7)	99 (17.6)	170 (30.3)	277 (49.4)	
Female	32 (8.9)	81 (22.4)	123 (34.1)	125 (34.6)	
Age					<0.001
18–29	33 (6.6)	119 (23.8)	126 (25.1)	223 (44.5)	
30–49	9 (2.7)	44 (13.3)	146 (44.1)	132 (39.9)	
50–59	3 (4.5)	15 (22.7)	14 (21.2)	34 (51.5)	
60+	2 (8.3)	2 (8.3)	7 (29.2)	13 (54.2)	
Nationality					0.321
Saudi	43 (5.2)	158 (19.2)	256 (31.1)	367 (44.5)	
Non-Saudi	4 (4.1)	22 (22.4)	37 (37.8)	35 (35.7)	
Region					0.233
Northern	0 (0.0)	3 (14.3)	11 (52.4)	7 (33.3)	
Eastern	3 (5.6)	10 (18.5)	13 (24.1)	28 (51.9)	
Western	9 (4.3)	43 (20.7)	67 (32.2)	89 (42.8)	
Middle	32 (5.3)	118 (19.5)	185 (30.6)	270 (44.6)	
Southern	3 (8.8)	6 (17.6)	17 (50.0)	8 (23.5)	
Blood type					0.094
O	72 (17.9)	21 (5.2)	121 (30.0)	189 (46.9)	
Non-O	92 (20.6)	18 (4.0)	148 (33.1)	189 (42.3)	
Unknown	16 (22.9)	8 (11.1)	24 (33.3)	24 (33.3)	
Chronic disease					0.484
Yes	12 (6.3)	34 (17.9)	67 (35.3)	77 (40.5)	
No	35 (4.8)	146 (19.9)	226 (30.9)	325 (44.4)	
Vaccine doses					0.028
One dose	31 (4.7)	130 (19.5)	200 (30.1)	304 (45.7)	
Two doses	15 (8.4)	39 (21.9)	55 (30.9)	69 (38.8)	
Three doses	1 (4.3)	6 (26.1)	10 (43.5)	6 (26.1)	
Four doses	0 (0.0)	3 (7.7)	21 (53.8)	15 (38.5)	
None	0 (0.0)	2 (11.8)	7 (41.2)	8 (47.1)	

## Data Availability

The data may be provided on reasonable request to the corresponding author.

## References

[1] Augustin M., Schommers P., Stecher M., Dewald F., Gieselmann L., Gruell H., Horn C., Vanshylla K., Di Cristanziano V., Osebold L. (2021). Post-COVID syndrome in non-hospitalised patients with COVID-19: A longitudinal prospective cohort study. Lancet Reg. Health Eur..

[2] Aldali J., Meo S.A., Al-Khlaiwi T. (2023). Adverse Effects of Pfizer (BioNTech), Oxford-AstraZeneca (ChAdOx1 CoV-19), and Moderna COVID-19 Vaccines among the Adult Population in Saudi Arabia: A Cross-Sectional Study. Vaccines.

[3] World Health Organization WHO Coronavirus (COVID-19) Dashboard. https://covid19.who.int/.

[4] Sehanobish E., Barbi M., Fong V., Kravitz M., Sanchez T.D., Asad M., Matsumura C., Ferastraoaru D., O’Neill M., Karagic M. (2021). COVID-19-Induced Anosmia and Ageusia Are Associated with Younger Age and Lower Blood Eosinophil Counts. Am J Rhinol Allergy..

[5] Díaz-Salazar S., Navas R., Sainz-Maza L., Fierro P., Maamar M., Artime A., Basterrechea H., Petitta B., Pini S., Olmos J.M. (2022). Blood group O is associated with post-COVID-19 syndrome in outpatients with a low comorbidity index. Infect. Dis..

[6] Moraschini V., Reis D., Sacco R., Calasans-Maia M.D. (2021). Prevalence of anosmia and ageusia symptoms among long-term effects of COVID-19. Oral Dis..

[7] Komal A., Noreen M., Akhtar J., Imran M., Jamal M., Atif M., Khan J., Roman M., Haq F.U., Aftab U. (2021). Analyses of ABO blood groups with susceptibility and symptomatic variations of COVID-19 infection, a questionnaire-based survey. Apmis.

[8] Paolo G. (2020). Does COVID-19 cause permanent damage to olfactory and gustatory function?. Med. Hypotheses.

[9] Mutiawati E., Fahriani M., Mamada S.S., Fajar J.K., Frediansyah A., Maliga H.A., Ilmawan M., Bin Emran T., Ophinni Y., Ichsan I. (2021). Anosmia and dysgeusia in SARS-CoV-2 infection: Incidence and effects on COVID-19 severity and mortality, and the possible pathobiology mechanisms—A systematic review and meta-analysis. F1000Research.

[10] Vaira L.A., De Vito A., Lechien J.R., Chiesa-Estomba C.M., Mayo-Yàñez M., Calvo-Henrìquez C., Saussez S., Madeddu G., Babudieri S., Boscolo-Rizzo P. (2021). New Onset of Smell and Taste Loss Are Common Findings Also in Patients with Symptomatic COVID-19 after Complete Vaccination. Laryngoscope.

[11] Rehman F.U., Omair S.F., Memon F., Rind B.J., Memon D.A., Ali S.A., Ahmed B., Ali N. (2021). The Relation of ABO Blood Group to the Severity of Coronavirus Disease: A Cross-Sectional Study From a Tertiary Care Hospital in Karachi. Cureus.

[12] Alabsi R.A.M., Sandeepa N.C., Misfer R.T., Alraqdi M.M., Hamdi M.I.M. (2022). Correlation between Post-COVID-19, Chemosensitive Function, Blood Group, and Oral Health-Related Quality of Life. Int. J. Dent..

[13] Al-Youha S.A., Alduaij W., Al-Serri A., Almazeedi S.M., Al-Haddad M., Jamal M.H., Shih A.W., Al-Sabah S.K. (2021). The impact of ABO blood groups on clinical outcomes and susceptibility to COVID-19: A retrospective study in an unselected population. Transfusion.

[14] Shesha N., Melebari S., Alghamdi S., Refaat B., Naffadi H., Alquthami K. (2022). Associations of Clinical Factors and Blood Groups with the Severity of COVID-19 Infection in Makkah City, Saudi Arabia. Front. Cell. Infect. Microbiol..

[15] Jawdat D., Hajeer A., Massadeh S., Aljawini N., Abedalthagafi M.S., Alaamery M. (2022). Correlation between ABO Blood Group Phenotype and the Risk of COVID-19 Infection and Severity of Disease in a Saudi Arabian Cohort. J. Epidemiol. Glob. Health.

[16] Ray J.G., Schull M.J., Vermeulen M.M.J., Park M.A.L. (2021). Association between ABO and Rh Blood Groups and SARS-CoV-2 Infection or Severe COVID-19 Illness. Ann. Intern. Med..

[17] Göker H., Aladağ-Karakulak E., Demiroğlu H., Ayaz C.M., Büyükaşik Y., Inkaya A.C., Aksu S., Sayinalp N., Haznedaroğlu I.C., Uzun Ö. (2020). The effects of blood group types on the risk of COVID-19 infection and its clinical outcome. Turk. J. Med. Sci..

[18] Rahim F., Amin S., Bahadur S., Noor M., Mahmood A., Gul H. (2020). ABO/Rh-D Blood types and susceptibility to Corona Virus Disease-19 in Peshawar, Pakistan. Pak. J. Med. Sci..

[19] Deschasaux-Tanguy M., de Edelenyi F.S., Druesne-Pecollo N., Esseddik Y., Allègre J., Srour B., Galan P., Hercberg S., Severi G., Zins M. (2023). ABO blood types and SARS-CoV-2 infection assessed using seroprevalence data in a large population-based sample: The SAPRIS-SERO multi-cohort study. Sci. Rep..

[20] Lesky E. (1973). Viennese serological research about the year 1900: Its contribution to the development of clinical medicine. Bull. N. Y. Acad. Med..

[21] Pereira E., Felipe S., de Freitas R., Araújo V., Soares P., Ribeiro J., dos Santos L.H., Alves J.O., Canabrava N., van Tilburg M. (2022). ABO blood group and link to COVID-19: A comprehensive review of the reported associations and their possible underlying mechanisms. Microb. Pathog..

[22] Zhu J., Wu C., Wu L. (2020). Associations between genetically predicted protein levels and COVID-19 severity. J. Infect. Dis..

[23] Kim Y., Latz C.A., DeCarlo C.S., Lee S., Png C.Y.M., Kibrik P., Sung E., Alabi O., Dua A. (2021). Relationship between blood type and outcomes following COVID-19 infection. Semin. Vasc. Surg..

[24] Cheng Y., Cheng Y., Cheng G., Chui C.H., Lau F.Y., Chan P.K.S., Ng M.H.L., Sung J.J.Y., Wong R.S.M. (2005). ABO Blood Group and Susceptibility to Severe Acute Respiratory Syndrome. JAMA.

[25] Wu Y., Feng Z., Li P., Yu Q. (2020). Relationship between ABO blood group distribution and clinical characteristics in patients with COVID-19. Clin. Chim. Acta.

[26] Goel R., Bloch E.M., Pirenne F., Al-Riyami A.Z., Crowe E., Dau L., Land K., Townsend M., Jecko T., Rahimi-Levene N. (2021). ABO blood group and COVID-19: A review on behalf of the ISBT COVID-19 Working Group. Vox Sang..

[27] Vaira L.A., Deiana G., Fois A.G., Pirina P., Madeddu G., De Vito A., Babudieri S., Petrocelli M., Serra A., Bussu F. (2020). Objective evaluation of anosmia and ageusia in COVID-19 patients: Single-center experience on 72 cases. Head Neck.

[28] Miwa T. (2022). Smell and Taste Dysfunctions Owing to COVID-19. Brain Nerves.

[29] Mahmud R., Rassel M.A., Monayem F.B., Sayeed S.K.J.B., Islam M.S., Islam M.M., Yusuf M.A., Rahman S., Islam K.M.N., Mahmud I. (2021). Association of ABO blood groups with presentation and outcomes of confirmed SARS CoV-2 infection: A prospective study in the largest COVID-19 dedicated hospital in Bangladesh. PLoS ONE.

[30] Badedi M., Darraj H., Alnami A.Q., Makrami A., Mahfouz M.S., Alhazmi K., Mahmoud N., Mosa H. (2021). Epidemiological and clinical characteristics of deceased COVID-19 patients. Int. J. Gen. Med..

